# Transient neonatal diabetes mellitus as an early diagnostic clue to *HNF1B*-related disease – two case reports and a literature review

**DOI:** 10.1186/s40348-026-00234-3

**Published:** 2026-04-16

**Authors:** Marcin Kołbuc, Paweł Bednarek, Rafał Motyka, Tomasz Jarmoliński, Marzena Michalak-Kloc, Bodo B. Beck, Małgorzata Urbańska-Kosińska, Marcin Zaniew

**Affiliations:** 1 Department of Pediatrics, University of Zielona Góra, ul. Zyty 28, Zielona Góra, 65-046 Poland; 2 Department of Pediatrics with Divisions of Pediatric Endocrinology, Diabetology, and Nephrology, University Hospital, Zielona Góra, Poland; 3 Department of Internal Medicine, University of Zielona Góra, Zielona Góra, Poland; 4 Department of Pediatrics and Nephrology, District Hospital, Międzyrzecz, Poland; 5 Department of Pediatric Bone Marrow Transplantation, Oncology and Hematology, Wroclaw Medical University, Wroclaw, Poland; 6 Department of Neonatology, University Hospital, Zielona Góra, Poland; 7 Institute of Human Genetics and Center for Molecular Medicine Cologne, Faculty of Medicine, University of Cologne, University Hospital Cologne, Cologne, Germany

**Keywords:** NDM, *HNF1B*, Hyperglycemia, Insulin, Deletion

## Abstract

**Background:**

Pathogenic variants in the *HNF1B* gene cause a multi

system disorder encompassing organ abnormalities—primarily affecting the kidneys and pancreas—as well as metabolic disturbances, collectively referred to as *HNF1B*-related disease. While maturity-onset diabetes of the young type 5 is a well-recognized manifestation, neonatal diabetes mellitus (NDM) associated with *HNF1B* is exceedingly rare, and has only been reported in patients harboring single nucleotide variants.

**Case presentation:**

We describe two unrelated female children presenting with transient NDM caused by a complete *HNF1B* gene deletion. Both developed hyperglycemia within the first days of life requiring short-term insulin therapy, followed by spontaneous normalization of glycemia. However, their subsequent phenotypes diverged significantly. The first patient exhibited bilateral renal dysplasia while maintaining normal neurodevelopment. In contrast, the second patient developed later-onset cystic kidney disease, neurodevelopmental delay, and dysmorphic features, consistent with a broader 17q12 deletion syndrome spectrum. Although, in both cases, kidney abnormalities and extra-renal features (NDM, hypomagnesemia, hyperuricemia) were observed, both patients experienced a delay in diagnosis. On follow-up, serial oral glucose tolerance tests (OGTT), HbA1c assessments, and glucagon stimulation tests to date demonstrated preserved β-cell function, with the exception of hyperglycemia in 30–60 min of the extended OGTT in one patient.

**Conclusions:**

These two cases represent the first report of transient NDM due to *HNF1B* deletion. Our findings broaden the molecular and clinical spectrum of *HNF1B*-related diabetes, and emphasize the importance of considering *HNF1B* defects in children with transient neonatal hyperglycemia.

**Supplementary Information:**

The online version contains supplementary material available at 10.1186/s40348-026-00234-3.

## Introduction

Patients with a defect in the *HNF1B* gene, known as *HNF1B-*related disease, may exhibit multi-organ involvement and a wide range of biochemical disturbances, and the condition may arise either from a pathogenic point alteration or a whole-gene deletion of *HNF1B* [1]. Typical manifestations include congenital anomalies of the kidney and urinary tract (CAKUT) and tubular dysfunction (e.g. hypomagnesemia, hypokalemia, and hyperuricemia), as well as extra-renal abnormalities such as pancreatic structural anomalies with endo- and exocrine functional impairment, urogenital malformations, hyperparathyroidism and defects of the skeletal system. In contrast to pathogenic single nucleotide variants, those patients with a complete *HNF1B* deletion as the part of 17q12 deletion syndrome (also including the following other genes *AATF*,* ACACA*,* C17ORF78*,* DDX52*,* DHRS11*,* DUSP14*,* GGNBP2*,* LHX1*,* MRM1*,* MYO19*,* PIGW*,* SYNRG*,* ADA2A*,* ZNHIT3*) may also present with neurocognitive disorders [2]. No clear genotype–phenotype correlation has been confirmed between point mutations and whole-gene deletions in terms of other organ manifestations. The selection of patients for molecular testing can be optimized using clinical tools such as the HNF1B score [3] or more recently developed machine learning–based predictive models estimating the probability of an *HNF1B* alteration [4].

In terms of glucose metabolism disorders, there is no known genotype-phenotype correlation in *HNF1B* disease, and patients may present throughout the entire pediatric age, starting from birth, as transient or permanent neonatal diabetes mellitus (NDM) [5] or glucosuria, elevated postprandial glucose, and marked glycemic variability [6] and, in subsequent years, as maturity-onset diabetes of the young type 5 (MODY5) [7, 8]. Another presentation is the new onset of diabetes after transplantation [9].

NDM is a monogenic disorder of glucose metabolism that occurs in neonates and infants under six months of age. Approximately 50% of cases are classified as transient NDM (TNDM), in which hyperglycemia resolves within the first few months of life, although recurrence may occur later in childhood or adolescence. In NDM, the most frequently identified molecular defects include imprinting anomalies on chromosome 6q24 (approximately two-thirds of cases) and activating mutations in *KCNJ11* or *ABCC8* [5].

Pathogenic variants in *HNF1B* are a rare cause of NDM [5, 10]. Previously reported cases have described patients requiring temporary insulin therapy for overt diabetes [11–14], as well as isolated instances of neonatal hyperglycemia [6]. To date, only pathogenic point variants in *HNF1B* have been reported in this clinical context. Here, we present two cases of TNDM associated with a complete *HNF1B* deletion, which expands the phenotypic and molecular spectrum of *HNF1B*-related disorders.

## Results

### Case 1

A female child from the first pregnancy was delivered at 35 weeks of gestation (premature rupture of membranes) with a birth weight of 2025 g (10–50th percentile). Due to respiratory failure, the neonate required non-invasive respiratory support until the second day of life. Hyperglycemia was noted from the first day (peak serum glucose, sGlu 298 mg/dL; peak serum insulin, sIns 35 µIU/mL ), requiring intensive insulin therapy until day 5, after which the glycemia gradually normalized. Laboratory tests also revealed transient hypomagnesemia (serum magnesium, sMg 0.65 mmol/L). Abdominal ultrasonography revealed bilateral renal dysplasia, and only the pancreatic head was visualized. A chest X-ray revealed the absence of the 12th pair of ribs. The family history was negative for diabetes and kidney disease. The mother had well-controlled Hashimoto’s thyroiditis. During the first year of life, the infant was regularly monitored at a diabetology out-patient clinic with no recurrence of glycemic abnormalities. She was discharged from the endocrinology/diabetology follow-up as no abnormalities were disclosed at the age of one year.

At the age of four years, an evaluation performed due to abdominal pain revealed mild hyperuricemia (serum uric acid, sUA 5.5 mg/dL) and elevated aspartate aminotransferase (AST 79.8 U/L). Other fasting parameters, including sMg, sGlu, glycated haemoglobin (HbA1c), C-peptide, and parathyroid hormone (PTH) levels remained within the reference ranges (0.79 mmol/L, 83 mg/dL, 5.07%, 1.53 ng/mL, and 18.21 pg/mL, respectively). Abdominal ultrasound revealed the pancreatic head and body, but not the tail. Given the constellation of renal and metabolic findings, *HNF1B* disease was suspected. Multiplex ligation-dependent probe amplification confirmed a complete *HNF1B* gene deletion (SALSA^®^ MLPA^®^ Probemix P241-E1 kit, MRC-Holland) – supplementary Fig. 1. Parental testing was negative, indicating a *de novo* variant.

**Fig. 1:**
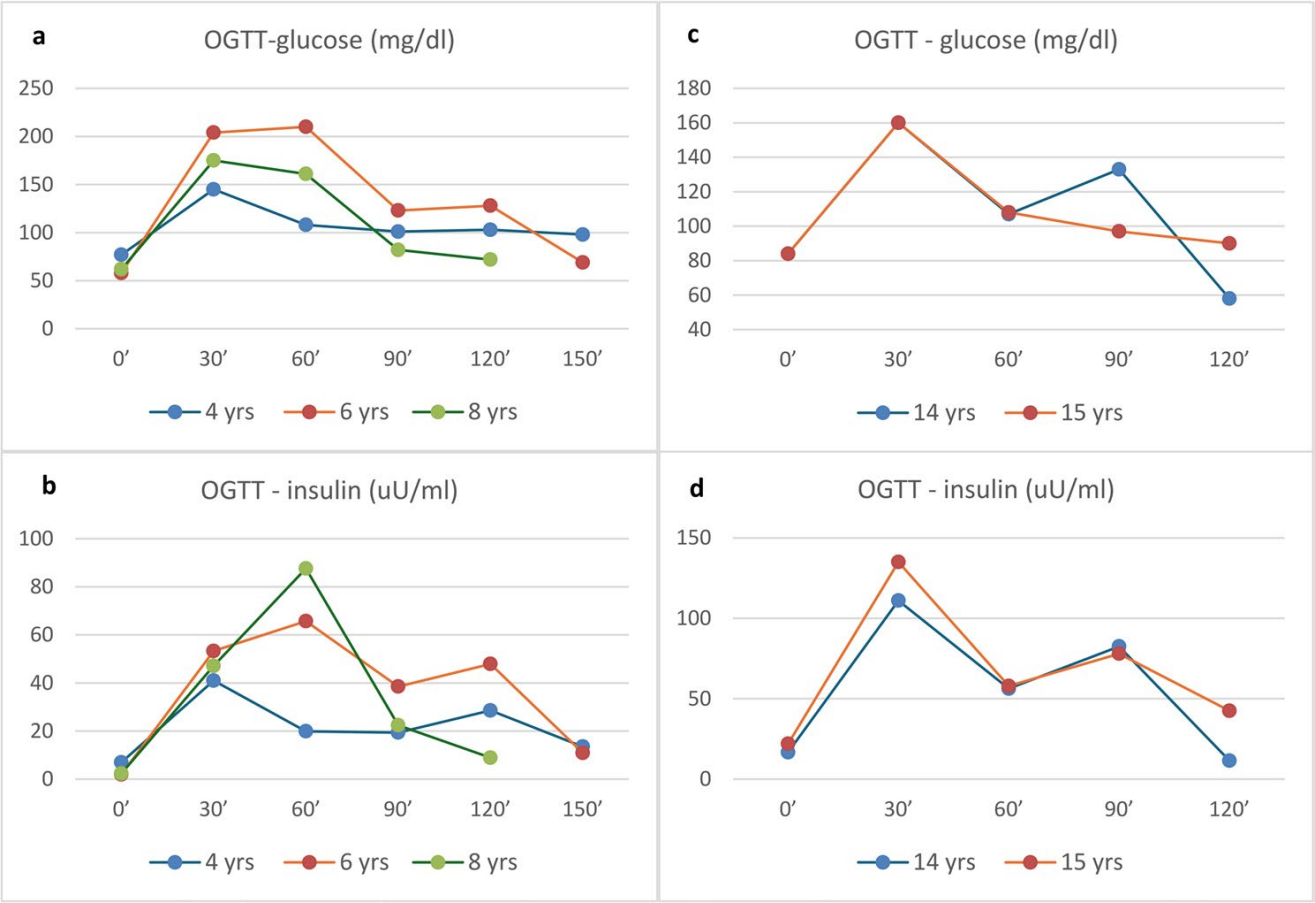
Blood glucose and insulin responses during an oral glucose tolerance test (OGTT) in the patient 1 (**a**-**b**) and patient 2 (**c**-**d**). Coloured lines correspond to the patient’s age at each visit

After confirming *HNF1B*-related disease, the patient underwent detailed diagnostic evaluations. An oral glucose tolerance test (OGTT) performed at 4 years of age, with glucose and insulin measured at 0–30–60–90–120 min, showed values within normal limits (Fig. 1a-b). In contrast, the follow-up OGTT at 6 years revealed a glucose increase > 200 mg/dL at 30 and 60 min (max. 210 mg/dL at 60 min). The subsequent OGTT at 8 years demonstrated a maximal glucose value of 175 mg/dL at 30 min. Glucose concentrations at 0 and 120 min remained within the diagnostic reference range. The HOMA index has consistently remained normal. HbA1c levels were also persistently within normal limits, with a maximum value of 5.32% at 4 years of age. C-peptide concentrations during the initial and subsequent assessments demonstrated an appropriate, at least two-fold increase following glucagon stimulation. Mild but persistent elevations in AST activity and hyperuricemia were observed, whereas sMg levels fluctuated between low and low-normal values. PTH concentrations were consistently normal. Total cholesterol, normal at 4 years, increased to 190 mg/dL at 6 years of age, with LDL-cholesterol at the upper limit of the reference range. Kidney ultrasound findings i.e. slightly increased bilateral echogenicity and small cysts in the left kidney remained stable over the years. Kidney function was preserved throughout the follow-up. Abdominal magnetic resonance imaging (MRI) performed at 8 years of age demonstrated bilateral kidney cysts, preserved corticomedullary differentiation, and a morphologically normal pancreas. The remaining clinical and biochemical data from the last follow-up visit are summarized in Table 1. The child is on Mg^2+^ supplementation since the age of six years.

**Table 1:** Summary of published cases of *HNF1B*-related neonatal diabetes mellitus

Parameter	Case 1	Case 2	
Age (years)	8	15	
Height-SDS	0.67	0.11	
BMI-SDS	-0.87	-1.6	

**Parameter**	**Reference values**	**Case 1**	**Case 2**
HbA1c (%)	4.8-6.0	4.8	5.24
Fasting glucose (mg/dl)	60–99	62	84
Fasting insulin (uU/ml)	3–17	2.36	22
Peptide C (ng/ml) 0 min/6min after glucagon inj (in test with glucagon- 0.5mg < 30 kg, 1mg > 30 kg)	-	1.23/2.53	3.54/8.19
sMg (mmol/l)	0.7–0.91	0.79^#^	**0.56** ^**#**^
FEMg (%)	< 5%	**7.24** ^**&**^	4.68
sCa (mmol/l)	2.15–2.55	2.6	2.5
FECa (%)	-	0.35	0.08
sP (mmol/l)	1.1-2.0	1.338	**1.048**
TmP/GFR (mmol/l)	1.15–2.44	1.19	**0.94**
sK (mmol/l)	3.5–4.5	4.1	3.8
sUA (mg/dl)	^*^2.5-5.0/^**^4.0-8.6	4.9	5.5
PTH (pg/ml)	15–65	34.1	**74.5**
ALT (U/l)	7–50	16	14.8
AST (U/l)	8–50	**81.2**	18.1
eGFR	> 90 ml/min	136.81	120.4

Abnormal values are shown in bold font

Abbreviations: *ALT* Alanine transaminase, *AST*, Aspartate aminotransferase, *BMI* Body mass index, *eGFR* estimated glomerular filtration rate (Schwartz formula, k = 0.413), *FECa*, Fractional excretion of Ca^2+,^*FEMg* Fractional excretion of magnesium, *HbA1c* Hemoglobin A1C, *PTH* Serum parathyroid hormone, *sCa* serum Ca^2+^, *SDS* Standard deviation score, *sK* serum potassium, *sMg* serum Mg^2+^, *sP* serum phosphate, *sUA* serum uric acid, *TmP/GFR* Tubular maximum of phosphate reabsorption over glomerular filtration rate

*sUA reference value for case 1; ** sUA reference value for case 2

# on Mg^2+^ supplementation

& this value is abnormal in the setting of hypomagnesemia (an increased Mg^2+^urinary excretion)

### Case 2

A female child was born prematurely at 31 weeks of gestation due to fetal compromise of unknown etiology, with a birth weight of 1320 g and body length of 40 cm (10–50th and 50th percentiles, respectively, according to Fenton’s preterm growth charts). The perinatal period was complicated by cardiorespiratory failure and necrotizing enterocolitis. On the third day of life, hyperglycemia (peak sGlu 249 mg/dL) was detected, requiring continuous insulin infusion, which was discontinued after five days after spontaneous normalization of glucose levels. Abdominal ultrasonography was unremarkable; however, the pancreas could not be visualized. The infant was discharged on day 42 of life with normal kidney function and normal serum electrolyte levels, apart from hypomagnesemia (sMg 0.72 mmol/l), according to pediatric reference ranges described by Ridefelt et al. [15]. The family history for kidney disease and diabetes was negative.

From two years of age, the patient required multidisciplinary follow-up due to a global developmental delay in the intellectual, motor, and speech domains. Epilepsy was suspected at the age of five. At six years, a single episode of fasting hyperglycemia (121 mg/dL) was documented, but not further investigated. From the age of seven years, she had been followed for hypothyroidism, hypercholesterolemia (max. 312 mg/dL), persistent metopic suture and scoliosis. Targeted next-generation sequencing (NGS) for familial hypercholesterolemia, covering *LDLR*,* APOB*,* PCSK9*, and *LDLRAP1* genes, revealed no pathogenic variants. At the age of ten years she had MRI of the spine, performed to evaluate scoliosis, but incidentally the study revealed bilateral kidney cysts. At 11 years of age, a comprehensive abdominal ultrasound confirmed cystic kidney disease with features consistent with a medullary sponge kidney, the pancreas was partially obscured by bowel gas; the portions that were visualized appeared sonographically homogeneous.

Given the kidney phenotype, she had NGS panel for cystic kidney disease, which unexpectedly showed a complete *HNF1B* deletion. A subsequent array comparative genomic hybridization (aCGH) confirmed the presence of 17q12 deletion – arr[GRCh37] 17q12 (34856055_36248918)x1, deletion size 1.4 Mb, which was compatible with the features experienced by the patient so far (supplementary Fig. 2). No pathogenic or likely pathogenic variants were identified in the remaining 44 genes included in the cystic kidney disease panel (*ANKS6*; *BICC1*; *CCDC41*; *CEP164*; *CEP290*; *COL4A1*; *CRB2*; *DCDC2*; *DNAJB11*; *DZIP1L*; *EYA1*; *GANAB*; *GLIS2*; *IFT172*; *INVS*; *IQCB1*; *JAG1*; *LRP5*; *MAPKBP1*; *MUC1*; *NEK8*; *NOTCH2*; *NPHP1*; *NPHP3*; *NPHP4*; *OFD1*; *PAX2*; *PKD1*; *PKD2*; *PKHD1*; *PRKCSH*; *RPGRIP1L*; *SDCCAG8*; *SEC61A1*; *Sect. 63*; *SIX5*; *TMEM67*; *TSC1*; *TSC2*; *TTC21B*; *UMOD*; *VHL*; *WDR19*; *ZNF423*). Only maternal genetic testing was available, which yielded a negative result.

During multiple follow-up hospitalizations between 12 and 14 years of age, persistent hypomagnesemia (0.63–0.66 mmol/l) accompanied by elevated PTH (131 pg/mL) and sUA levels (5.5–6.4 mg/dL) were observed. High total cholesterol levels (211–290 mg/dL) with very high LDL-cholesterol (186 mg/dl) persisted, triglycerides remained within reference values and high-density lipoprotein (HDL) was elevated. The OGTT (0-30-60-90-120 min) remained within normal limits, maximal glucose value of 160 mg/dL at 30 min (Fig. 1c-d). For the first time, mild facial dysmorphism (high forehead, narrow eyelid fissures) and digital anomalies (clinodactyly and low-set thumbs) were noted. A repeated electroencephalogram demonstrated background activity faster than expected for her age, but no epileptiform discharges were recorded. A lipid-lowering therapy (rosuvastatin) and oral Mg^2+^ supplementation was initiated.

At the most recent follow-up visits at 15 years of age, a computed tomography revealed calcifications within the renal pyramids of both kidneys, more prominent and extensive on the left side, consistent with medullary nephrocalcinosis (NC). Additionally, both kidneys showed parenchymal cysts. In the MRI scan, the pancreas was only partially developed, with the head and a rudimentary body visible, previously not recognized in ultrasound examinations. Hyperparathyroidism persisted, accompanied for the first time by hypophosphatemia with renal phosphate wasting, reflected by phosphaturia and low TmP/GFR. Persistent hypomagnesemia was observed, despite oral supplementation. An evaluation for metabolic causes of nephrolithiasis and NC did not disclose any other abnormalities. With respect to lipid profile, total cholesterol and LDL normalized during statin treatment (175 mg/dl and 88 mg/dl, respectively), very low-density lipoproteins and triglycerides were normal, HDL was elevated. Thyroid peroxidase antibodies and thyroglobulin antibodies were negative. HbA1c and glucose concentration in OGTT were very similar to the previous year (Fig. 1c-d), but fasting insulin, and consequently, HOMA-IR index were higher i.e. 4.83 compared to 3.46 at a year before, and to that value of 2.49 obtained 2 years ago. Metformin treatment was recommended. The remaining clinical and biochemical data are summarized in Table 1.

## Discussion

This study reports two cases of TNDM caused by a complete deletion of the *HNF1B*, addressing a gap in current knowledge regarding this molecular etiology. Unlike previously described cases of *HNF1B*-NDM, which were typically associated with intrauterine growth restriction and insulin deficiency, our patients presented with birth weight appropriate for gestational age, highlighting greater metabolic and potentially allelic heterogeneity than previously recognized.

Here, we highlight the need for considering of *HNF1B* disease in the setting of NDM. Neither of our patients was timely diagnosed despite some classical features of *HNF1B* disease. Unfortunately, NDM in our patients was not recognized as an important feature, which caused lack of follow-up visits, and a delay in diagnosis. Of note, in case 1, a combination of NDM and kidney dysplasia should have prompted the presence of *HNF1B* defect already in the neonatal period, even if the coexistence of diabetes and kidney anomalies has limited predictive value. In a cross-sectional study based on the Polish Monogenic Diabetes Registry, Sztormwasser et al. reported that the detection rate of *HNF1B* mutations in this cohort did not exceed 30% [16]. The penetrance of diabetes among individuals carrying causative *HNF1B* variants is incomplete, and strongly age-dependent, ranging from 36**%** in pediatric populations [17], 60% in mixed population [18] to nearly 80% in adults [19]. Unfortunately there are no data on a detection rate of *HNF1B* in children with NDM and kidney disease, as this combination has been rarely reported so far. NDM is a subtype of monogenic diabetes that occurs within the first six months of life. Most cases are caused by chromosomal aberrations at 6q24 or pathogenic variants of *KCNJ11* or *ABCC8* [5]. To date, only four cases of NDM associated solely with *HNF1B* alterations have been reported [11–14]. In three cases, patients required short-term insulin therapy in the first month of life; in two of these cases, diabetes relapsed in the first decade of life. One patient required intermittent insulin therapy from the 15th day of life and permanent treatment from the age of six. Pathogenic *HNF1B* variants can also cause neonatal glycemic disturbances that do not meet the criteria for NDM. Iafusco et al. described a newborn with CAKUT carrying a *de novo* heterozygous pathogenic *HNF1B* variant who exhibited glucosuria, elevated postprandial glucose, and marked glycemic variability [6]. A summary of the key clinical features of all known *HNF1B*-NDM cases is shown in Table 2.

**Table 2:** Summary of published cases of HNF1B-related neonatal diabetes mellitus

Author/study	HNF1B variant	SGA/AGA	Insulin requirement	Diabetes relapse	Kidney phenotype	Pancreatic anomalies	Other systemic features
Pezzino et al. [14]	splice site variant c.1045 + 1G > A (*de novo*)	AGA	Required from day 1; discontinued within 1 month	No	Left kidney cysts	Pancreatichypoplasia, hyperechogenicity	↑LFT
Yorifuji et al. [11] (index sib)	missense variant c.443 C > G; p.Ser148Trp (*de novo*)	SGA	Insulin therapy from day 15, intermittent until 6 y, permanent thereafter	No	Slightly small right kidney, two cysts	No structural abnormality	Epilepsy, DD
Beckers et al. [13]	frameshift variant c.499_504delinsCCCCT; p.Ala167fs (*de novo*)	SGA	48 h insulin during parenteral feeding	Yes (at 5 y)	Unilateral kidney agenesis, cystic kidney	Pancreatic atrophy, exocrine insufficiency	Cholestasis, ↑LFT
Edghill et al. [12]	missense c.443 C > T; p.Ser148Leu (*de novo*)	SGA	Required at 17th day of life, discontinued within 6 days	Yes (at 8 y)	Kidney dysplasia	Pancreatic atrophy, mild exocrine insufficiency	↑LFT
This study (case 1)	deletion (*de novo*)	AGA	Required for the first 5 days of life	No	Bilateral kidney dysplasia	Normal pancreas	↑LFT, ↓sMg, ↑CHOL, ↑sUA, Absence of the 12th pair of ribs
This study (case 2)	17q12 deletion (mother neg.)	AGA	Required from the 3rd to 8th day of life	No	Bilateral kidney cysts, medullary sponge kidney	Pancreas hypoplasia (head and rudimentary body)	↓sMg, ↑sUA, ↑PTH, ↑CHOL, facial dysmorfism, epilepsy, DD, digital anomalies, ↓sP, ↑sIns

Abbreviations: *AGA* Appropriate for gestational age, *DD* Developmental dela, *SGA* Small for gestational age

↑PTH hyperparathyroidism; ↑sUA, hyperuricemia; ↑CHOL, hypercholesterolemia; ↑sIns, hyperinsulinism; ↑LFT, elevated liver function tests; ↓sMg, hypomagnesemia; ↓sP, hypophosphatemia

Two mechanisms have been proposed to underlie *HNF1B*-NDM. First, it involves the underdevelopment of pancreatic β-cells and functional insulin deficiency, leading to impaired fetal growth (small for gestational age, SGA) [5, 12]. Observations from Kotalová et al. further support this concept [20]. In their comprehensive review of the liver phenotype in *HNF1B* disease, the authors summarized 12 reported cases of cholestasis and identified pancreatic anomalies in 75% of affected individuals. Moreover, six of the nine patients with available birth data were born SGA, and all subsequently developed diabetes. These findings reinforce the hypothesis that both pancreatic maldevelopment and fetal growth restriction may play a central role in the pathogenesis of diabetes in *HNF1B*-related disorders. No genotype–phenotype correlations were observed. Further evidence for the lack of a clear genotype–phenotype correlation in *HNF1B*-related disease comes from the study by Craven et al. who, in a single-center analysis of diabetes and endocrine manifestations, reported diabetes in patients carrying both intragenic alterations and whole-gene deletions, irrespective of birth weight status [21]. In fact, ¾ of known *HNF1B*-NDM cases presented with pancreas atrophy/hypoplasia, all were SGA, 3 out of 4 were found to have elevated liver enzyme levels. Our patients were born with birth weights appropriate for gestational age (AGA, both 10–50th percentile). In the first our case, elevated liver transaminase levels were observed, whereas in the second case, pancreatic structural anomaly was present.

The second mechanism results from hepatic insulin resistance. Pearson et al. compared diabetes phenotypes due to *HNF1B* and *HNF1A* alterations and showed that patients harbouring *HNF1B* alterations have hyperinsulinemia and associated dyslipidemia consistent with insulin resistance, and may have a different β-cell defect [22]. In our first patient, monitored from birth to 8 years of age, no insulin resistance or hyperinsulinism has been identified to date. In the second case, we do observe progressive insulin resistance; however, this concerns a patient who has been under our care only since the age of 12 years with pre-existing hypercholesterolemia and a positive family history in this regard. Additionally, the HOMA index was assessed when the patient entered puberty, a period in which insulin resistance physiologically appears. Her HOMA-IR index has increased with age, being < 3 at 10 years and reaching 4.83 at 15 years.

The complex etiology of NDM in patients with *HNF1B* pathogenic variants may, at least in part, reflect the pleiotropic effects of disease causing *HNF1B* alterations or the contribution of additional genetic or environmental modifiers. Yorifuji et al. described two siblings harbouring the same heterozygous *HNF1B* pathogenic variant, yet exhibiting markedly discordant phenotypes [11]. One child presented with CAKUT limited to a slightly small right kidney with 2 cysts and permanent NDM, the only case reported to date. The sibling displayed bilateral multicystic kidneys and hyperechogenic right kidney, reaching end-stage kidney failure at 2 years of age without any glucose metabolism abnormalities. Functional studies performed by the authors confirmed that the S148W mutation leads to decreased *GLUT2* transcriptional activity, which may explain the permanent NDM observed in one sibling, similarly to pathogenic variants in the *SLC2A2* gene causing Fanconi–Bickel syndrome. Building on these results, Gong et al. demonstrated that two novel likely pathogenic *HNF1B* variants, E105K and G454R, identified in five patients with MODY5 exert the opposite effect on *GLUT2* transcription compared to S148W [23]. These findings highlight that different *HNF1B* variants can have distinct functional effects, providing an explanation of phenotypic differences in glucose metabolism disorders in *HNF1B* alterations.

Interestingly, Yorifuji et al. [11] and Edghill et al. [12] independently reported patients carrying distinct amino acid substitutions (S148L and S148W, respectively) affecting the same *HNF1B* residue (S148), supporting the functional importance of this site. Notably, however, phenotypic outcomes differed substantially, i.e. Edghill’s patient developed transient rather than permanent neonatal diabetes, while another carrier described by Yorifuji showed no neonatal diabetes. This striking discrepancy suggests the coexistence of an additional pathogenic variant in another gene implicated in β-cell function, such as *KCNJ11*, which acts as a second molecular hit necessary for the development of diabetes [24]. Unfortunately, our patients were not tested for other genetic causes of NDM.

To the best of our knowledge, no cases of NDM resulting from *HNF1B* gene deletions have been reported. The youngest patients described in the literature with *HNF1B* deletions and disturbances in glucose metabolism were 33 and 22 months old, both exhibiting a phenotype consistent with MODY5 [8]. These individuals presented with elevated HbA1c levels and preserved endogenous insulin secretion, as reflected by normal C-peptide concentrations, and were successfully managed through dietary modification alone. Our patients did not experience recurrence of diabetes during the follow-up period. Serial glucagon stimulation tests and OGTT remained within normal limits, and the HbA1c values were consistently normal. The transient hyperglycemia exceeding 200 mg/dL at 30 and 60 min of the OGTT observed in one of our patients likely represents an early manifestation of developing diabetes, despite normal glucose concentrations at 0 and 120 min. Similarly, this phenomenon was observed in another patient [25]. The clinical course observed in our patients is consistent with the hypothesis that carbohydrate metabolism disturbances secondary to progressive β-cell dysfunction emerge gradually, most commonly manifesting after the age of 14 years.

In terms of NDM pathogenesis in our patients, this process may be influenced by both structural pancreatic abnormalities and intrinsic β-cell dysfunction associated with *HNF1B* alterations. Under conditions of increased metabolic demand, such as perinatal stress, a reduced pancreatic functional reserve may predispose to transient neonatal hyperglycemia. Titchiner et al. [26] evaluated the frequency of insulin use for hyperglycemia in neonates admitted to neonatal intensive care units; among nearly 30,000 neonates included (gestational age 22–32 weeks), insulin therapy was required in 24% of cases. Identified risk factors included lower gestational age and birth weight, prolonged hospitalization, and the need for mechanical ventilation. Several of these factors were present in patient 2. This supports the hypothesis that a reduced β-cell functional reserve in patients with *HNF1B* deletions may increase susceptibility to hyperglycemia requiring insulin therapy in critically ill neonates, beyond any direct transcriptional effects of haploinsufficiency. In contrast, patient 1 lacked these perinatal risk factors, requiring only non-invasive respiratory support, with no evidence of pancreatic malformation, an appropriate birth weight for gestational age, and currently normal glucose metabolism. A shared feature with previously reported NDM cases is the presence of elevated liver enzymes, representing the mildest hepatic phenotype of *HNF1B* disease. As noted in the review by Kotalova et al., this phenotype is frequently observed in individuals who subsequently develop MODY5 [20]. Continued follow-up of patient 1 is therefore warranted to monitor for the potential development of MODY5, which would further support underlying β-cell dysfunction.

Importantly, in addition to glycemic disturbances, our patients also exhibited biochemical abnormalities suggestive of *HNF1B* disease, most notably hypomagnesemia. Since the publication by Adalat et al. [27], the relationship between pathogenic *HNF1B* variants and hypomagnesemia has gained increasing attention. The authors showed that low sMg is more frequently observed in patients with CAKUT carrying *HNF1B* mutations compared to those without such alterations. Mechanistically, *HNF1B* regulates transcription of the *FXYD2* gene, which encodes the gamma subunit of the Na⁺/K⁺-ATPase involved in tubular magnesium and calcium transport. Pathogenic variants of *HNF1B* reduce *FXYD2* expression, resulting in renal magnesium wasting and hypocalciuria. Importantly, hypomagnesemia is a typical hallmark of *HNF1B* disease, and could serve as a sensitive marker, yet frequently underestimated predictor of *HNF1B* mutations [28]. In both our patients, hypomagnesemia was an early feature, yet remained unrecognized, and its recognition at that stage might have substantially accelerated the diagnostic process. In this regard, the selection of candidates for molecular testing should be guided by dedicated clinical tools, such as the HNF1B score [3] or predictive models [4], with particular attention to the use of appropriate age-dependent reference ranges for sMg.

With respect to hypocalciuria, in patient 2 it was accompanied by hypophosphatemia with renal phosphate wasting, reflected by phosphaturia and low TmP/GFR, most likely related to hyperparathyroidism. Elevated PTH promotes renal phosphate wasting and may also favour calcium–phosphate deposition within the kidney parenchyma, thereby contributing to NC despite hypocalciuria. However, HNF1B cohorts do not routinely assess serum phosphate and report hypophosphatemia/phosphaturia as a result of hyperparathyroidism to fully support this mechanism. In addition, the kidney phenotype of medullary sponge kidney, observed in this patient, is intrinsically associated with NC, suggesting that the kidney calcifications likely reflect a combination of metabolic disturbances related to hyperparathyroidism and the underlying structural kidney abnormality.

In conclusion, this report expands the phenotypic and molecular spectrum of *HNF1B*-related disease. NDM should prompt consideration of *HNF1B* defects, especially in the setting of kidney or magnesium abnormalities, which may allow for timely diagnosis, and personalized care of children with this rare disease.

## Supplementary Information

Supplementary Material 1: Supplementary Figure 1. MLPA analysis demonstrating a heterozygous deletion of HNF1B exon 1 to 9 and unremarkable CNV analyses for GCK, HNF1A, and HNF4A in the index patient (a). MLPA analysis performed in patient‘s mother (b) and father (c) was unremarkable. Supplementary Figure 2. Visualisation of the 60k aCGH result in CytoGenomics software (Agilent). The upper image shows a schematic representation of probe signals across all chromosomes and highlights the deletion on chromosome 17, marked with a black arrow. The medium image shows the chromosome 17 ideogram, with the minimal chromosomal coordinates for the 17q12 deletion, encompassing 45 probes and 1.39 Mb. The left bottom image shows a closer view of the deleted region of chromosome 17, marked with a black arrow, while the right bottom image shows the maximum deletion size (1.52 Mb), including the gene content.

## Data Availability

The data used to write this report is available from the corresponding authoron reasonable request.
