# Supplementary material for: Transient neonatal diabetes mellitus as an early diagnostic clue to HNF1B-related disease – two case reports and a literature review
Source: Mol Cell Pediatr. 2026 Apr 16;13:20. doi: 10.1186/s40348-026-00234-3 (PMC13083689; doi:10.1186/s40348-026-00234-3)

**Supplementary Figure 1.** MLPA analysis demonstrating a heterozygous deletion of *HNF1B* exon 1 to 9 and unremarkable CNV analyses for *GCK*, *HNF1A*, and *HNF4A* in the index patient (a). MLPA analysis performed in patient‘s mother (b) and father (c) was unremarkable.


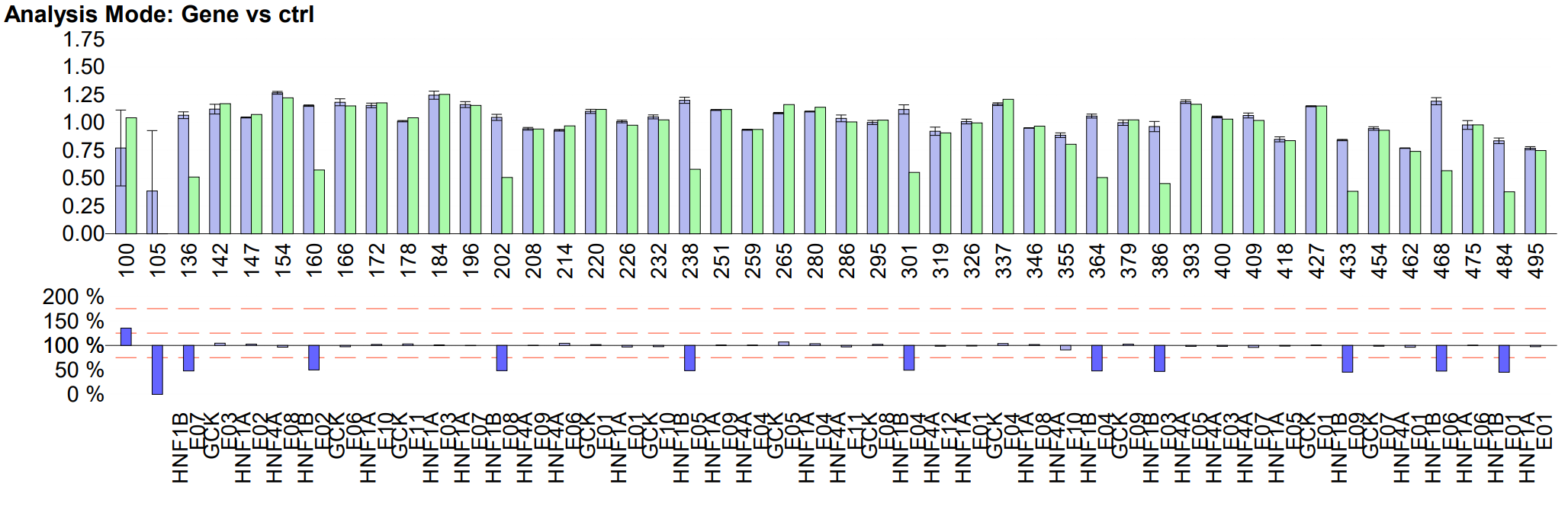


**a**


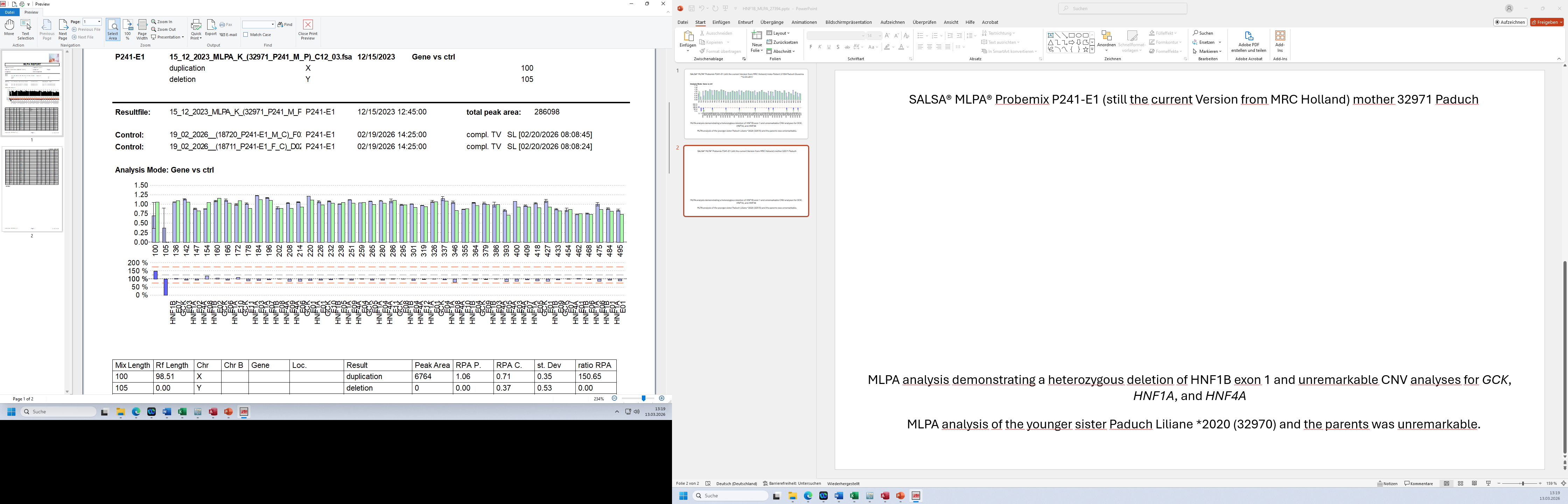


**b**


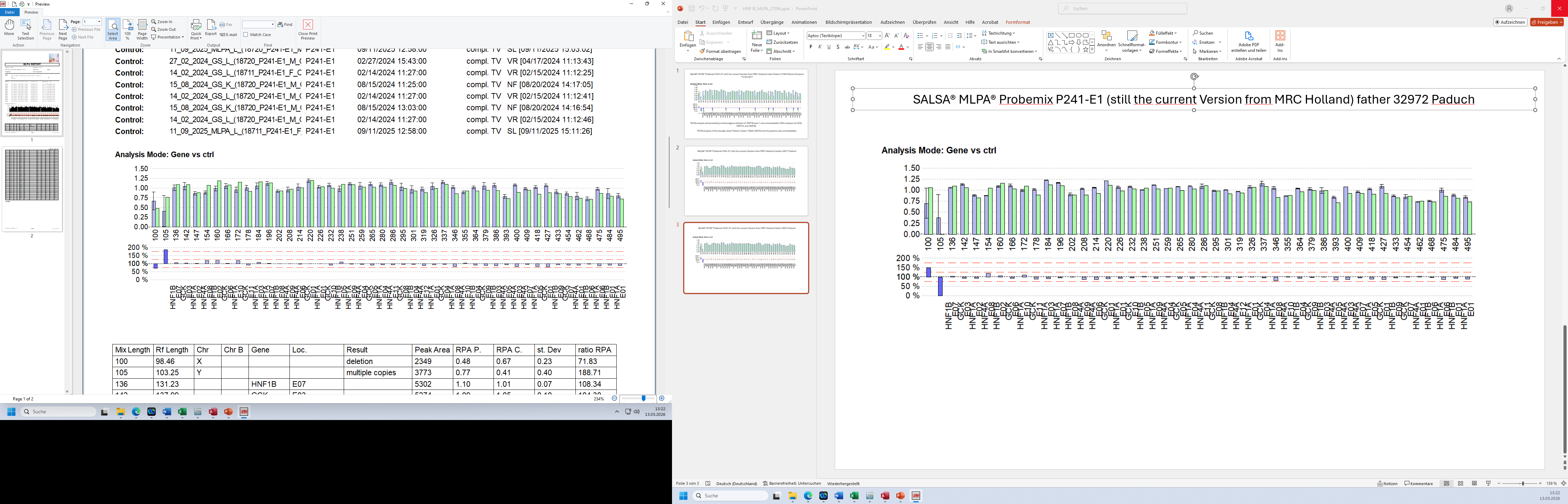


**c**

**Supplementary Figure 2.** Visualisation of the 60k aCGH result in CytoGenomics software (Agilent). The upper image shows a schematic representation of probe signals across all chromosomes and highlights the deletion on chromosome 17, marked with a black arrow. The medium image shows the chromosome 17 ideogram, with the minimal chromosomal coordinates for the 17q12 deletion, encompassing 45 probes and 1.39 Mb. The left bottom image shows a closer view of the deleted region of chromosome 17, marked with a black arrow, while the right bottom image shows the maximum deletion size (1.52 Mb), including the gene content.


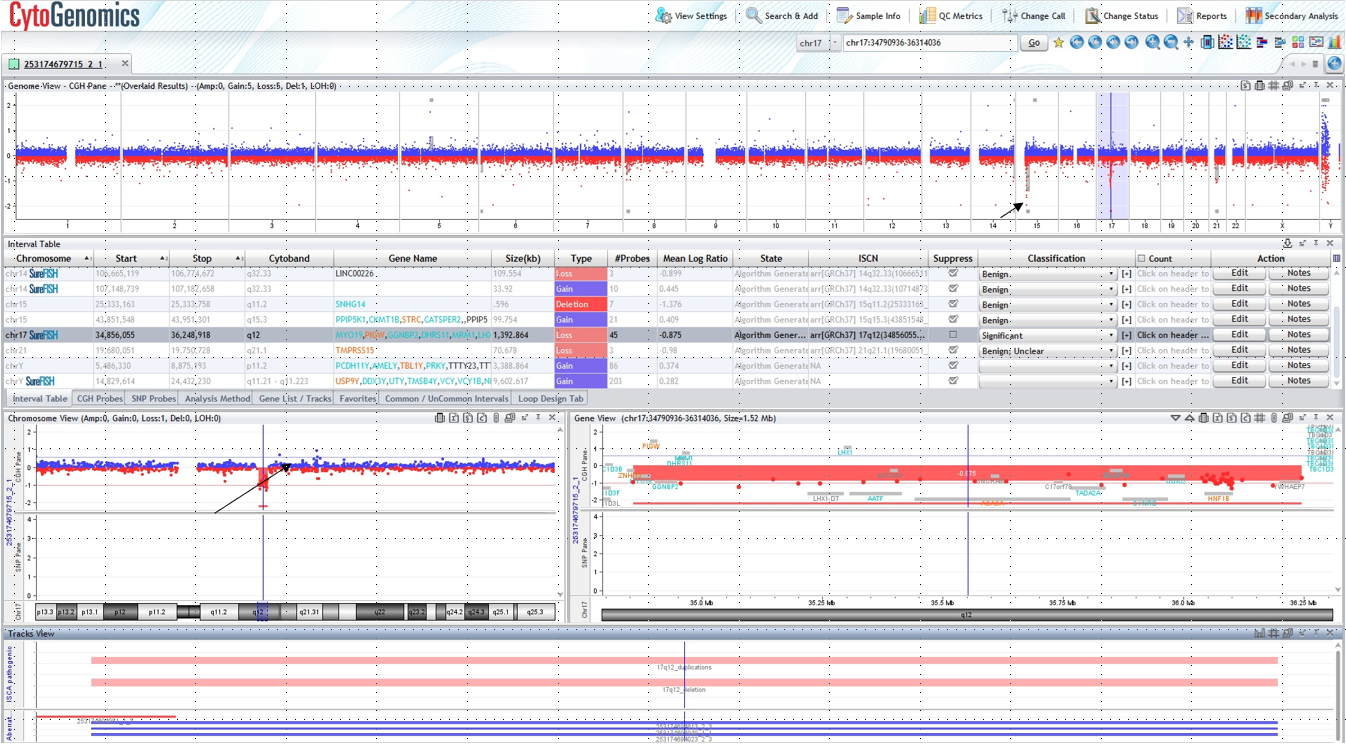

Supplement: Supplementary file 1 — Supplementary Material 1: Supplementary Figure 1. MLPA analysis demonstrating a heterozygous deletion of HNF1B exon 1 to 9 and unremarkable CNV analyses for GCK, HNF1A, and HNF4A in the index patient (a). MLPA analysis performed in patient‘s mother (b) and father (c) was unremarkable. Supplementary Figure 2. Visualisation of the 60k aCGH result in CytoGenomics software (Agilent). The upper image shows a schematic representation of probe signals across all chromosomes and highlights the deletion on chromosome 17, marked with a black arrow. The medium image shows the chromosome 17 ideogram, with the minimal chromosomal coordinates for the 17q12 deletion, encompassing 45 probes and 1.39 Mb. The left bottom image shows a closer view of the deleted region of chromosome 17, marked with a black arrow, while the right bottom image shows the maximum deletion size (1.52 Mb), including the gene content. [file 40348_2026_234_MOESM1_ESM.docx]
